# Distinct temporal features of genuine and deliberate facial expressions of surprise

**DOI:** 10.1038/s41598-021-83077-4

**Published:** 2021-02-09

**Authors:** Shushi Namba, Hiroshi Matsui, Mircea Zloteanu

**Affiliations:** 1 Psychological Process Team, BZP, Robotics Project, RIKEN, Kyoto, 6190288 Japan; 2 Center for Human-Nature, Artificial Intelligence, and Neuroscience, Hokkaido University, Hokkaido, 0600808 Japan; 3 Department of Criminology and Sociology, Kingston University London, Kingston Upon Thames, KT1 2EE UK

**Keywords:** Psychology, Human behaviour

## Abstract

The physical properties of genuine and deliberate facial expressions remain elusive. This study focuses on observable dynamic differences between genuine and deliberate expressions of surprise based on the temporal structure of facial parts during emotional expression. Facial expressions of surprise were elicited using multiple methods and video recorded: senders were filmed as they experienced genuine surprise in response to a jack-in-the-box (Genuine), other senders were asked to produce deliberate surprise with no preparation (Improvised), by mimicking the expression of another (External), or by reproducing the surprised face after having first experienced genuine surprise (Rehearsed). A total of 127 videos were analyzed, and moment-to-moment movements of eyelids and eyebrows were annotated with deep learning-based tracking software. Results showed that all surprise displays were mainly composed of raising eyebrows and eyelids movements. Genuine displays included horizontal movement in the left part of the face, but also showed the weakest movement coupling of all conditions. External displays had faster eyebrow and eyelid movement, while Improvised displays showed the strongest coupling of movements. The findings demonstrate the importance of dynamic information in the encoding of genuine and deliberate expressions of surprise and the importance of the production method employed in research.

## Introduction

Facial expressions are regarded as affective signals which can convey social information about an expresser's experience of an emotional event^1,2^. However, not all facial displays are a result of genuine emotional experiences. Some expressions are produced intentionally and are deceptive in their signaling^3,4^. Genuine displays occur when a sender experiences actual genuine affect, while deliberate displays reflect the strategic intent of the sender and may not reflect or be absent of congruent underlying affect.

Prior research has shown that people (i.e. *decoders*) are not very good at determining if an expression is genuine or deliberate^5,6^. This poor performance may be due to a lack of reliable markers that can distinguish veracity^7^ and senders’ ability to produce genuine-looking expressions with little effort^8^, enough to fool others^9^. However, while humans may be unable to make accurate emotional authenticity discriminations, it is important to investigate if genuine and deliberate displays do have morphological and dynamic differences that can be measured using more advanced technology-based approaches. Recent work has, indeed, indicated that spontaneous and posed displays can be differentiated, to an extent, using advanced approaches. For example, Namba et al.^10^ recorded spontaneously genuine facial reactions to emotion-eliciting films and deliberately posed expressions by asking participants to intentionally express four emotions: surprise, amusement, disgust, and sadness. Analyses of these expressions revealed appearance-based differences between spontaneous and posed facial displays. The results clarified the morphologic differences for disgust and the dynamic differences for amusement and surprise, but also indicated that these differences were not always clear on the type of emotion.

Past research investigating emotional displays has focused strongly on the facial muscle activation occurring during an emotional event using manual coding approaches, such as the Facial Action Coding System (FACS)^11^, describing each facial action on an anatomical basis (i.e. Action Unit (AU)). However, the nature of facial expressions is that they are dynamic in presentation. Previous studies indicated that dynamic displays improve coherence in the identification of affect, leads to more accurate emotion judgments^12^, and, importantly, more accurate emotional authenticity discrimination^8,13^. Nevertheless, the role of facial movement in the discrimination of genuine displays remains an elusive topic.

Typically, research considering expression dynamics has employed a data-driven approach, rooted in perceptual emotion categorization of observers^14,15^. An alternative approach to investigating how facial displays unfold over time is an automatic sampling of the configuration of points (landmarks) from expressions. Under this approach, instead of providing an output of AUs based on a photo frame or a sequence of frames, the sampling landmarks themselves are outputted as they appear, avoiding information loss and ensuring higher time-resolution than the manual FACS coding. This operationalization is important, as it avoids the strong, and potentially unfounded, assumptions of specific AUs or facial configuration being representative of emotional authenticity (i.e. the *reliable muscles* hypothesis^16^). As such, we propose that investigations of differences between spontaneously felt and deliberately posed emotional displays should adopt an elicitation-based approach instead of an appearance-based approach^17,18^.

Zloteanu et al.^19^ also indicated that using the umbrella term “posed (or deliberate)” to describe all non-genuine expressions obfuscates different production methods for generating deliberate displays. For example, van der Schalk et al.^20^ produced posed displays to match the activation of specific AU combinations related to emotion categorization, while Namba et al.^10^ filmed posed displays following senders being instructed to express the emotional words. In fact, Zloteanu et al.^19^ has shown that deliberate displays where senders focused on their outward expression are perceived as more genuine-looking than deliberate displays where senders focused on their affective feeling. Since observers’ responses can vary depending on the type of deliberate displays, the encoding aspects of these should be considered. Among several facial displays representing cross-culturally well-recognized emotion categories (e.g., happiness, sadness, anger, fear, disgust)^21^, surprise has peculiar function as an emotion^22,23^. It can convey information about an expectancy discrepant event and capture the perceiver’s attention^24–26^.

Theoretical and empirical accounts suggest that the surprise expression is associated with eyebrow (AU1 + 2) and eyelid (AU5) movements, although variations can occur^1,10^. The opening of the mouth (AU25) was also included in the stereotypical surprise expressions, but it has not been reliably found to represent surprise^27,28^. The cognitive-evolutionary model proposes that surprise is evoked by unexpected and schema-discrepant events^29,30^. Much prior work shows that unexpected events can elicit facial part activations that favor raising the eyebrow and/or eyelid^27,28,31–33^. The activations of the upper face around the eyebrow and eyelid can be considered as main components of the genuine surprise expressions because the subcortical system related to spontaneous responses tends to more strongly affect these upper face muscles^34,35^.

To avoid misleading inferences regarding the genuine-deliberate facial display dimension, research on the encoding of facial displays should clarify how the production method employed to generate the expressions may create differences in appearance. Zloteanu et al.^8,19^ have already recorded four different deliberate surprise displays as well as spontaneous surprise displays: Spontaneous surprise display in response to a jack-in-the-box, which can be described as the unexpected and schema-discrepant eliciting event, was filmed (Genuine condition). This manipulation is justified as Ekman et al.^36^ suggest that the surprised expressions are produced by the unexpectedness of such an event. Deliberate surprise displays were recorded with no preparation (Improvised condition), by mimicking the outward expression in the Genuine condition (External condition), or after having first experienced genuine surprise themselves (Rehearsed condition). This research revealed that the different surprise displays impacted decoders’ inferences. Although genuine surprise displays were perceived as the most genuine, there were also differences between deliberate displays in Improvised and Rehearsed conditions. In addition, dynamic presentation, compared to static, improved authenticity discrimination accuracy and perceptual differences between expressions. Accordingly, it can be assumed that there are perceptual dynamic differences both between spontaneous and deliberate, but also between different deliberate expressions. Thus, elucidating encoding aspects of these recorded displays with dynamic information shed light on research for facial expressions.

Although research to investigate how these surprise displays unfold from the encoding aspect was restricted, Namba et al.^10^ revealed the sequential differences of facial action between genuine and deliberate surprise displays. This research showed that genuine surprise showed the raising eyelid movement (AU5) occurred earlier than the raising eyebrow movement (AU1 + 2), while deliberate surprise showed both eyebrow and eye movements simultaneously (AU1 + 2 + 5). However, this finding has been investigated using manual facial action coding, and thus an automatic sampling of the configuration of points (landmarks) from facial expressions might be better for uncovering fine-grained dynamic features of facial expression including follow-follower relationship between eyebrow and eyelid movements.

Taken together, it remains unclear how spontaneous felt surprise displays are different from deliberately posed surprise displays in terms of spatio-temporal properties. The differences between deliberate displays elicited using different production methods also remain elusive. The current study, thus, focused on the core facial movement (i.e., eyebrow and eyelid movements) in surprise expressions under Genuine, Improvised, External, and Rehearsed conditions recorded in the two studies by Zloteanu et al.^8,19^. In the current study, the moment-to-moment *x* and *y* coordinates of eyelids and eyebrows in the 127 videos of surprised reactions were annotated with deep learning-based tracking software (‘DeepLabCut’). The extracted coordinates were used to investigate temporal structure or topological features of face parts, which were expected to differentiate genuine surprise displays from deliberate, or even between different deliberate displays. More specifically, it was predicted that raising movements of the eyebrows and eyelids composed all surprise displays and that the eyelid movements preceded eyebrow movements in genuine surprise displays, consistent with Namba et al.^10^. Furthermore, according to Zloteanu et al.^8,19^, it was assumed that the different deliberate displays will also differ from each other but be overall more similar than with genuine displays; the exploratory nature of the study does not permit directional hypotheses for these differences.

## Methods

### Participants

127 participants (115 females, 12 males; *M*_Age_ = 22.69, *SD* = 4.13) were recruited through the University’s SONA system for online recruitment. Ethics approval was provided for all aspects of the experiment by University College London’s Department of Psychology Ethics Committee (CPB/2013/009). Informed consent was obtained from all participants. All participants were video-recorded under one of the following four conditions: Genuine, Improvised, Rehearsed, and External. All data is fully HIPAA-compliant and was handled in accordance with the Data Protection Act 1998 and the tenets of the Declaration of Helsinki.

### Procedures

An overview of the stimuli creation process is provided below. The full procedure is described in the previous studies^8,19^. The camera in all conditions was arranged at eye-level and recorded participants from the beginning of the startle reactions through the end of these responses.

In the Genuine condition, participants sat in front of the jack-in-the-box and turned the wheel until the toy jumped out, prompted by a melody. The exact functioning of the toy was never described to participants, nor was the target emotion ever explicitly mentioned.

In the Improvised condition, participants turned the wheel like those in the Genuine condition, but the toy’s mechanism was disconnected and did not jump out. Instead, they watched a video on a tablet placed in front of the jack-in-the-box. The video was counting down while providing the same timing and melody used in the Genuine condition. When “NOW” appeared on the screen, participants had to feign that they were surprised. They were not told how to act surprised, simply to display a convincing portrayal.

In the Rehearsed condition, at first, participants experienced how the jack-in-the-box works, as did the Genuine condition. After, they performed the same actions as the Improvised condition but were instructed to recreate their surprised reaction when the word “NOW” appeared. The difference between Rehearsed and Improvised conditions was whether participants have an experience of how the jack-in-the-box functions.

In the External condition, participants watched a randomly selected video from the Genuine condition and were told to copy what they saw. Their task was similar to the Improvised condition but were told to mimic the behavior they had seen in the video when the word “NOW” appeared.

### Facial movement tracking

**Figure 1:**
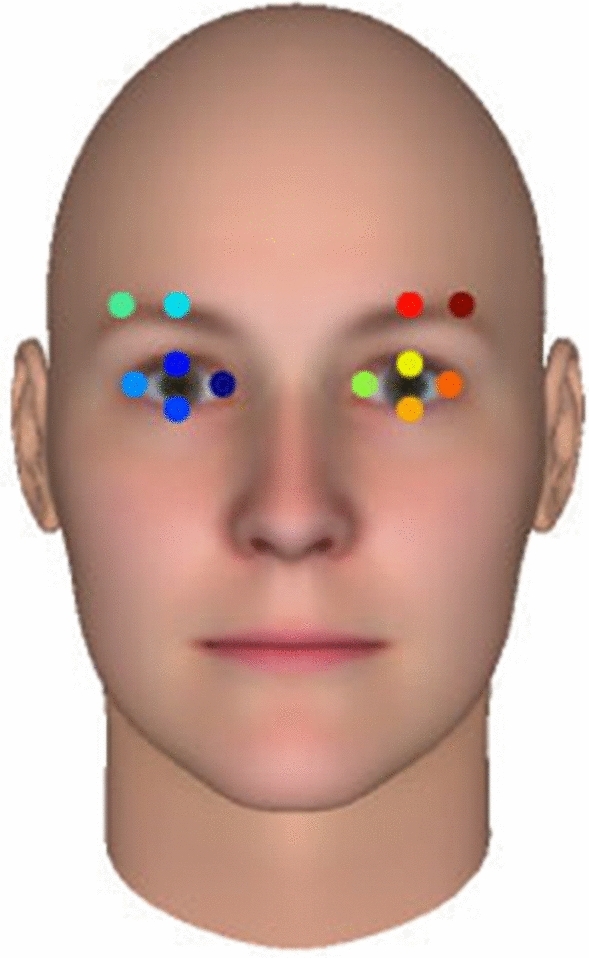
The annotated points of facial expression that this study focused on: upper and right (left) sides of the eyebrow, right (and left) outer corners of the eye, inner corners of the eye, and top and bottom of the eyelid.

All reactions were video-recorded with a Panasonic SDR-T50 camcorder, set at 1920 × 1080 pixels and 25 fps. Tracking was performed by python-based tracking software (“DeepLabCut”)^37,38^. Two-dimensional *x–y* coordinates of facial parts (Fig. 1) were extracted frame-by-frame. The landmarks were captured on every time points ranging from − 1000 ms to + 1000 ms from the onset of facial expressions. One female participant in Rehearsed condition was excluded because she completely covered her face with her hands while reacting to the “NOW” signal on the tablet, resulting in 126 participants: Genuine condition = 32 (31 females, 1 male), Improvised condition = 31 (28 females, 3 males), External condition = 32 (27 females, 5 males), Rehearsed condition = 31 (28 females, 3 males).

The *x* and *y* coordinates of all 12 tracking points were used, resulting in 24 tracking data. All tracking points were processed with smooth spline function to remove noise before analysis. Given that our main interest was focused on the topological features and their dynamics, but not other characteristics such as size, orientation of faces, distance from the jack-in-the-box, and so on. Using generalized Procrustes analysis^39^, these two-dimensional data can be registered as landmark configurations into optimal registration for multivariate statistical analysis of facial shape. Specifically, to standardize the size and orientation of each landmark, represented by a set of 12 tracking points, we normalized the 2D datasets based on centroid size and superimposed them using a least-squares method, which is a common technique in the field of morphometrics^40,41^.

### Statistical analysis

The temporal-topological configuration of each facial part consists of a high-dimensional space (in our case, twelve facial parts, *x* and *y* coordinates, resulting in 24 dimensions). Interpreting high-dimensional data is often challenging, and thus, one would need to extract the low-dimensional features from it using a dimensionality reduction technique^42^. To differentiate genuine and deliberate surprise using the temporal and topological features of facial parts, spatial components for surprised responses among the four conditions were extracted using Independent Component Analysis, which is used for dimensionality reduction (ICA)^43^. The FastICA algorithm was used to extract the topological features of facial parts^43^.

Based on the ICA results—which can be considered as a form of clustering—the current study focused on the *y* coordinates of the top of the eyelids and the upper sides of the eyebrows. To avoid noise from including head and body movements, the *y* coordinates of both the eyebrows and the eyelids were subtracted from the *y* coordinates of the inner corners of the eyes.

As for investigating temporal features further, we calculated the speed of eyebrows and eyelids by 3^rd^ order approximation. We further explored the differences for speed between conditions using one-way between-subjects ANOVAs to compare the effect of the four expression conditions (Genuine, Improvised, External, and Rehearsed) on the speed of each vertical movement. Furthermore, we assessed temporal coupling between eyelids and eyebrows. The cross-correlation approach allows the quantification of the synchrony or follow-follower relationship of two behavioral time series.

All analyses were performed using R statistical software, version 3.4 (https://www.r-project.org/), alongside ‘BayesFactor’, ‘dlcpr’, ‘fastICA’, ‘shapes’, and ‘tidyverse’ packages^44–47^.

## Results

### Distinct topological features of genuine surprise compared to deliberate expressions

**Figure 2:**
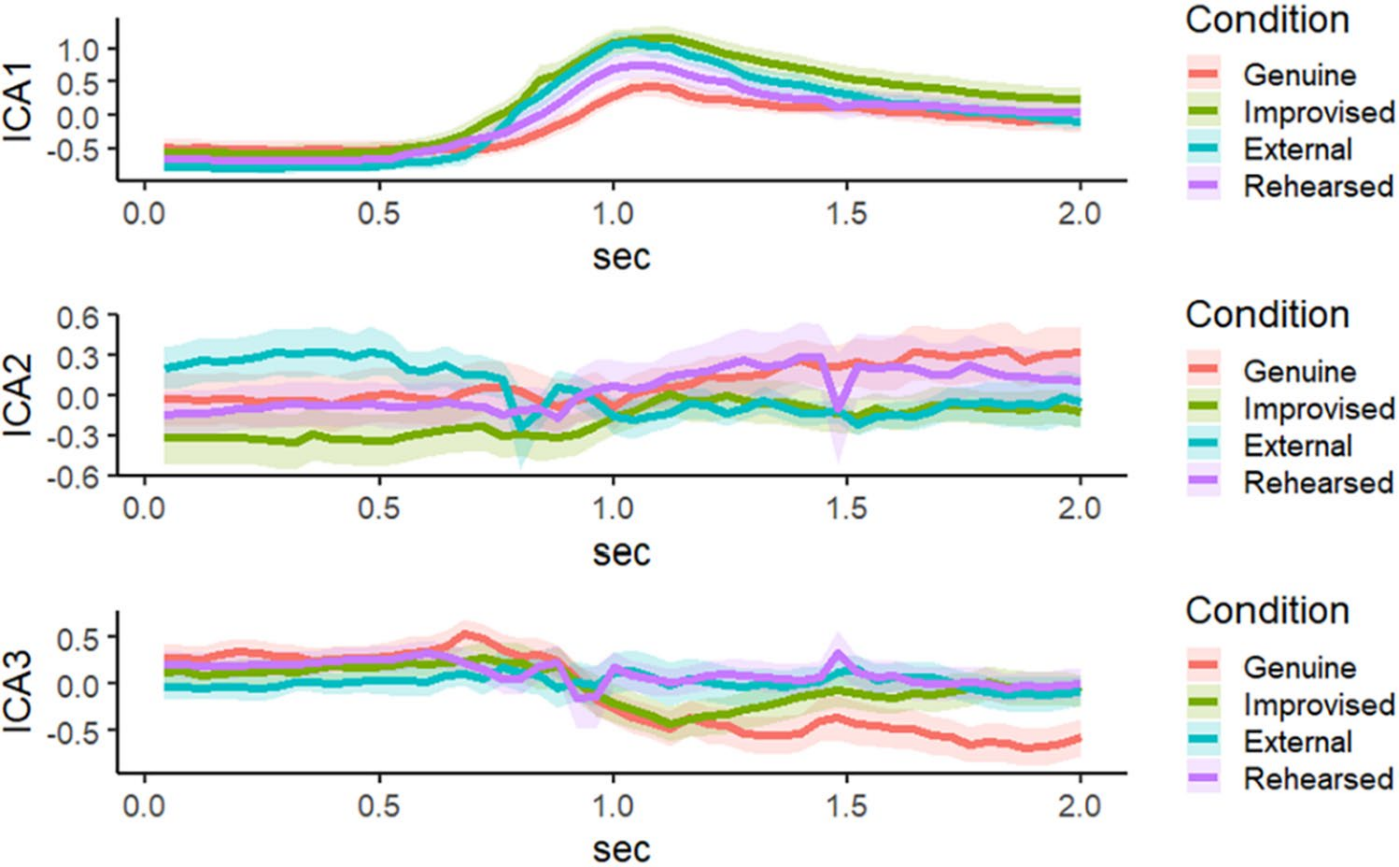
Independent Component among the four expression conditions. The ribbons represent ± 1 standard error.

**Figure 3:**
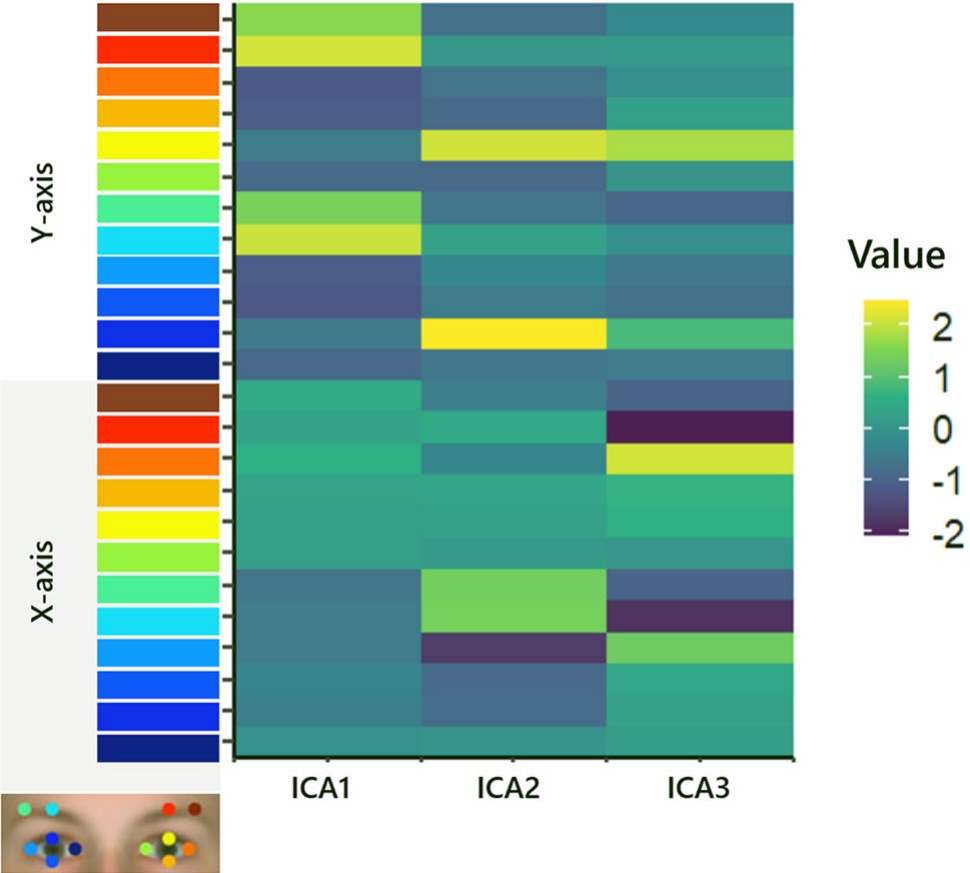
Heatmap of loadings for each Independent Component. Value colors represent the contribution of each facial part on independent component scores. Component 1 corresponded to raising the eyebrows (Loading mean for the center part of the right eyebrow = 2.07; Loading mean for the center part of the left eyebrow = 2.14). Component 2 is the y-movement of both eyelids (Loading mean for the upper part of the right eyelid = 2.48; Loading mean for the upper part of the left eyelid = 2.13). Component 3 represents the x-movement of the left eye and eyebrow (Loading mean for the outer part of the left eye = 2.14, Loading mean for the center part of the left eyelid = -2.07).

ICA was used to provide spatial components for all four conditions. ICA can provide insight into which tracking data contributed most to the facial dynamics. Figure 2 shows three independent components for surprised responses per condition. The detail of which facial annotated tracking data contributed to each component is described in Fig. 3. To be concise, we discuss the two highest independent component scores contributing to each low-dimensional component.

By inspecting the relative contribution of each facial part to the independent components, the former two components (i.e., Component 1 and Component 2) suggest that *y*-movement of eyelids and eyebrow components were the main contributors of all surprise expressions based on loading compared to other movements. The results of Component 3 and visual inspection of Fig. 2 indicated that the left parts of genuine displays might be vulnerable to horizontal movements. Therefore, we focused on the dynamic information for raising both eyelids and eyebrows, which may also contribute to form each surprise display.

As a reference for future research, we conducted a similar analysis for the mouth parts, reported in the Supplemental Information, as prior research on surprise has suggested the role of the mouth features in interpreting different types of surprise^48^. The supplemental analysis uncovered that the mouth-opening size is smaller in the Genuine condition, compared to only the Improvised condition. However, given our current predictions, we focus only on the relationship between the eyebrow and the eyelid.

### The speed of raising the eyebrow and the eyelid

**Figure 4:**
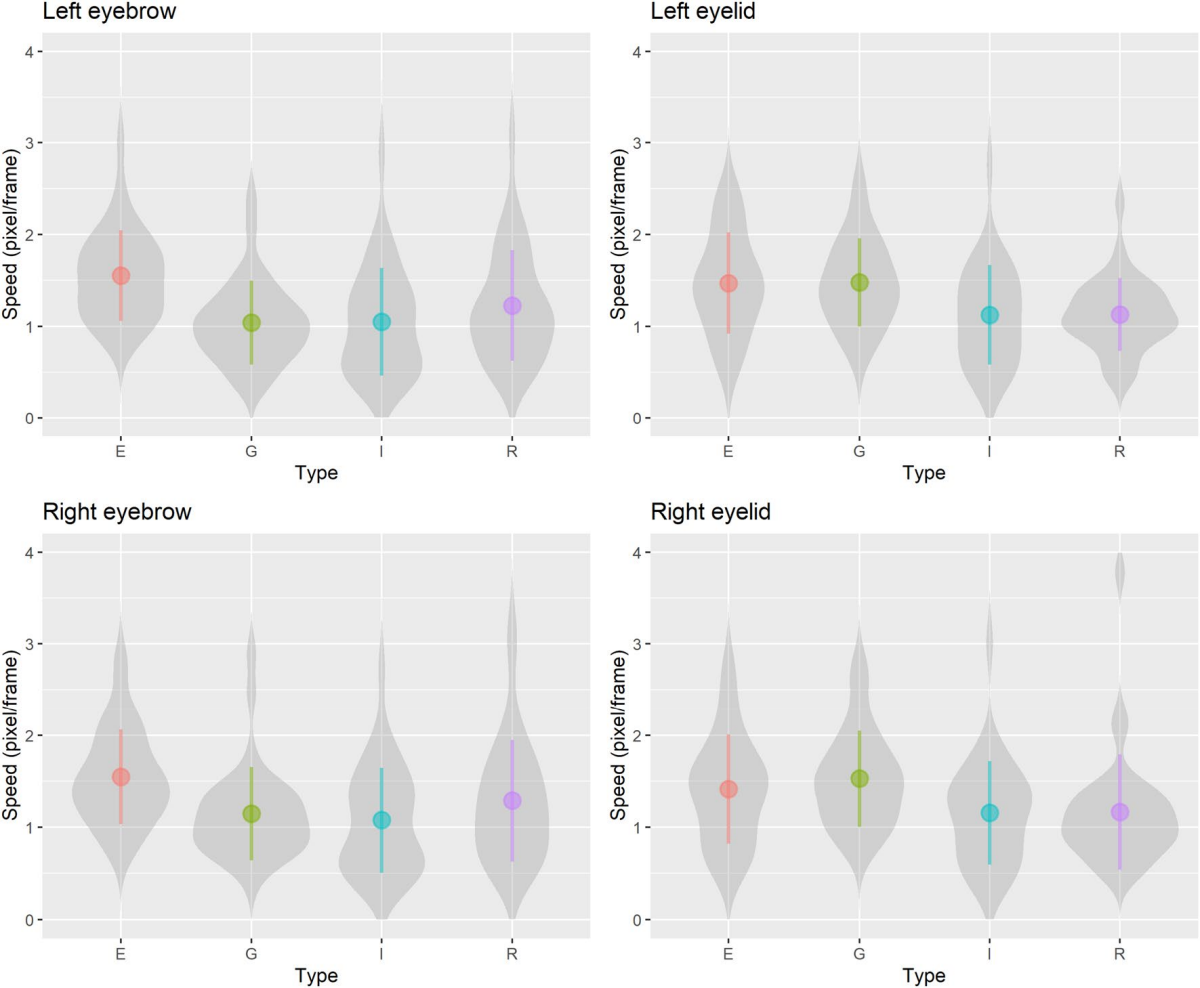
The speed (pixel/frame) of the vertical movement for both eyebrow and eyelid. Violin plots show the Kernel probability density of the data. Each point indicates the mean and the bars show 1 standard deviation range.

The dynamic information in the facial displays serves to discriminate the perceptual differences between spontaneous and deliberate displays, as suggested in prior work^8,13,49^. To understand the role of dynamic information in discrimination and perception, we further investigate the features which differentiate genuine surprise from the different types of deliberate surprise. Figure 4 shows the speed of the vertical movement for both eyebrow and eyelid.

We further explored the differences for speed (pixel/frame) between conditions using one-way between-subjects ANOVAs, comparing the effect of expression condition on the speed of each vertical movement. Levene’s test confirmed that equal variances can be assumed in all ANOVAs, all *p*s > 0.14. For the left eyebrow, there was a significant effect of expression conditions, *F*(3, 122) = 6.31, *p* < 0.001, ${\eta }_{G}^{2}$ = 0.13, *JZS BF*_*10*_ = 55.38 (against intercept only model). Specifically, multiple comparison using Shaffer’s modified sequentially rejective Bonferroni procedure showed that the vertical movement of the eyebrow in the External condition was faster than in the Genuine, *t*(64) = 3.81, *p* < 0.002, Hedge’s *g* [*95%CI*] = 0.94 [0.43, 1.45], and Improvised condition, *t*(63) = 3.71, *p* < 0.002, Hedge’s *g* = 0.92 [0.41, 1.44]. Furthermore, the left eyelid also showed a significant effect based on expression conditions, *F*(3, 122) = 5.36, *p* = 0.002, ${\eta }_{G}^{2}$ = 0.12, *JZS BF*_*10*_ = 18.90. Multiple comparison supported that the vertical movement of the eyelid in the External condition was faster than in the Rehearsed, *t*(63) = 2.90, *p* < 0.006, Hedge’s *g* = 0.72 [0.22, 1.23], and Improvised condition, *t*(63) = 2.67, *p* = 0.010, Hedge’s *g* = 0.66 [0.16, 1.17]. Genuine condition expressions were also faster than in the Rehearsed, *t*(63) = 3.16, *p* < 0.003, Hedge’s *g* = 0.79 [0.28, 1.29], and Improvised condition, *t*(63) = 2.73, *p* < 0.009, Hedge’s *g* = 0.68 [0.18, 1.18].

On the right side of the face, there was a significant effect of expression conditions on eyebrow movement, *F*(3, 122) = 4.32, *p* = 0.007, ${\eta }_{G}^{2}$ = 0.10, *JZS BF*_*10*_ = 5.83. Similar to the results of left eyebrow, multiple comparison showed that the vertical movement of the eyebrow in the External condition was faster than in the Genuine, *t*(64) = 2.84, *p* < 0.016, Hedge’s *g* = 0.70 [0.20, 1.20], and Improvised condition, *t*(64) = 3.31, *p*s < 0.008, Hedge’s *g* = 0.82 [0.32, 1.33]. For the right eyelid there was a significant effect of expression conditions, *F*(3, 122) = 3.34, *p* = 0.022, ${\eta }_{G}^{2}$ = 0.08, but the Bayes Factor did not strongly support the evidence, *JZS BF*_*10*_ = 1.90, and multiple comparisons did not show any significant differences, *t*s < 2.34, *p*s > 0.127, Hedge’s *g*s < 0.58.

In sum, the speed of both eyebrows and eyelids when mimicking other facial expressions (i.e., External condition) was generally higher than in other conditions.

### Decreased temporal coupling in genuine surprise

Next, we explored the coupling between raising eyebrows and eyelids. As an index of the temporal features of two eyelid and eyebrow movements, temporal coupling between raising eyelids and eyebrows were analyzed with a cross-correlation analysis that can quantify the follow-follower relationship or synchrony of two time-series data.

**Figure 5:**
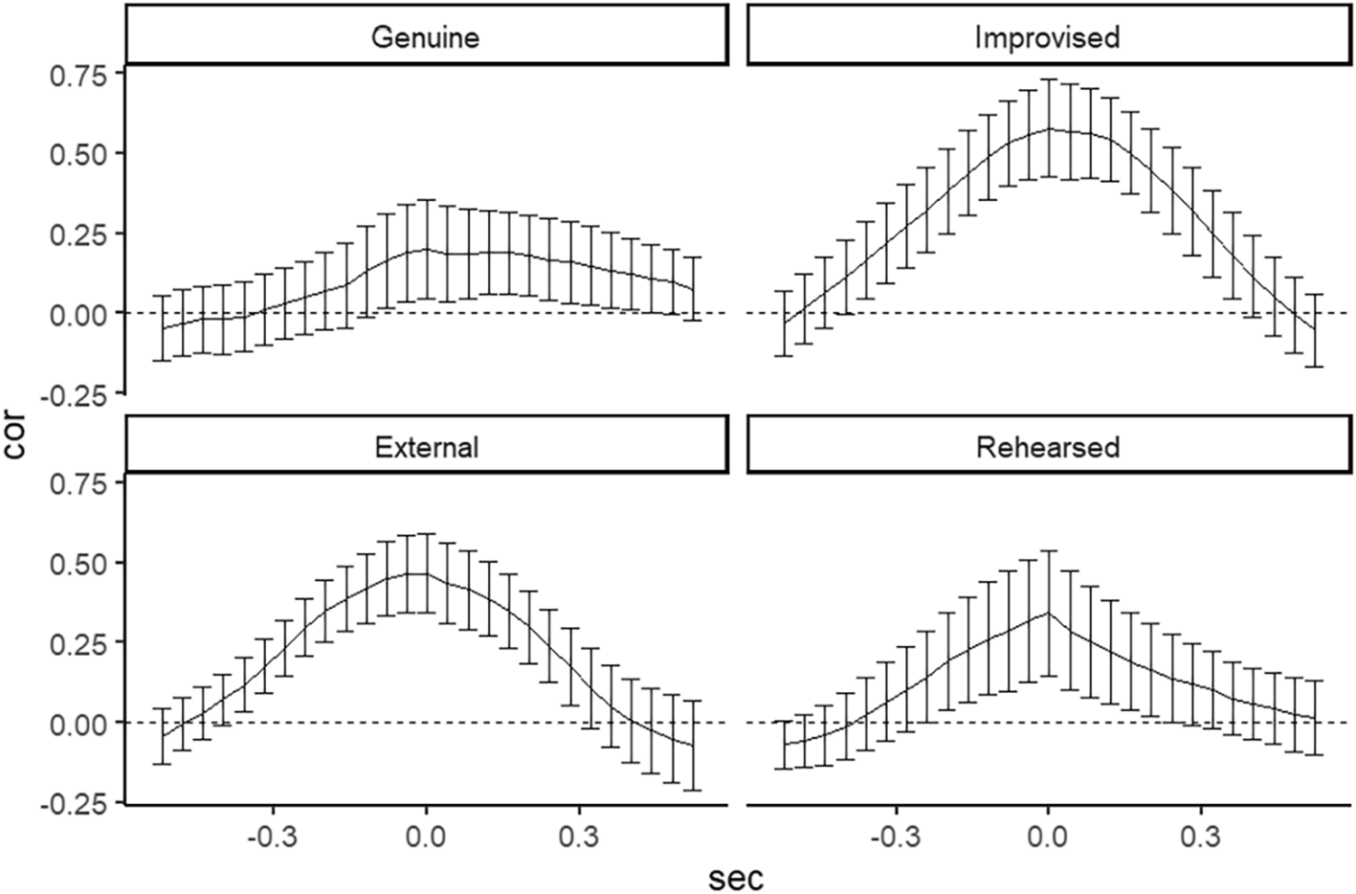
The cross-correlations between the left eyebrow and left eyelid for the four conditions. Error bar represents 95% Confidence Intervals.

For left parts of facial displays, shown in Fig. 5, all conditions revealed significant cross-correlations in terms of 95% confidence intervals. The result that all cross-correlations at the lag 0 ms have been highest than other lag times can be considered as no follow-follower relationships between eyebrow and eyelid. Furthermore, there were medium couplings in the Improvised (*r* = 0.58, *SD* = 0.41, *95%CI* = [0.42, 0.73]) and External (*r* = 0.47, *SD* = 0.35, *95%CI* = [0.34, 0.59]) conditions beyond zero at the lag 0 ms. Whereas the pattern of coupling in the Genuine (*r* = 0.20, *SD* = 0.43, *95%CI* = [0.05, 0.36]) and Rehearsed (*r* = 0.34, *SD* = 0.53, *95%CI* = [0.15, 0.54]) conditions indicated a lower cross-correlations than other conditions. This indicated that genuine displays on the left side have especially small coupling between eyelid and eyebrow movements.

**Figure 6:**
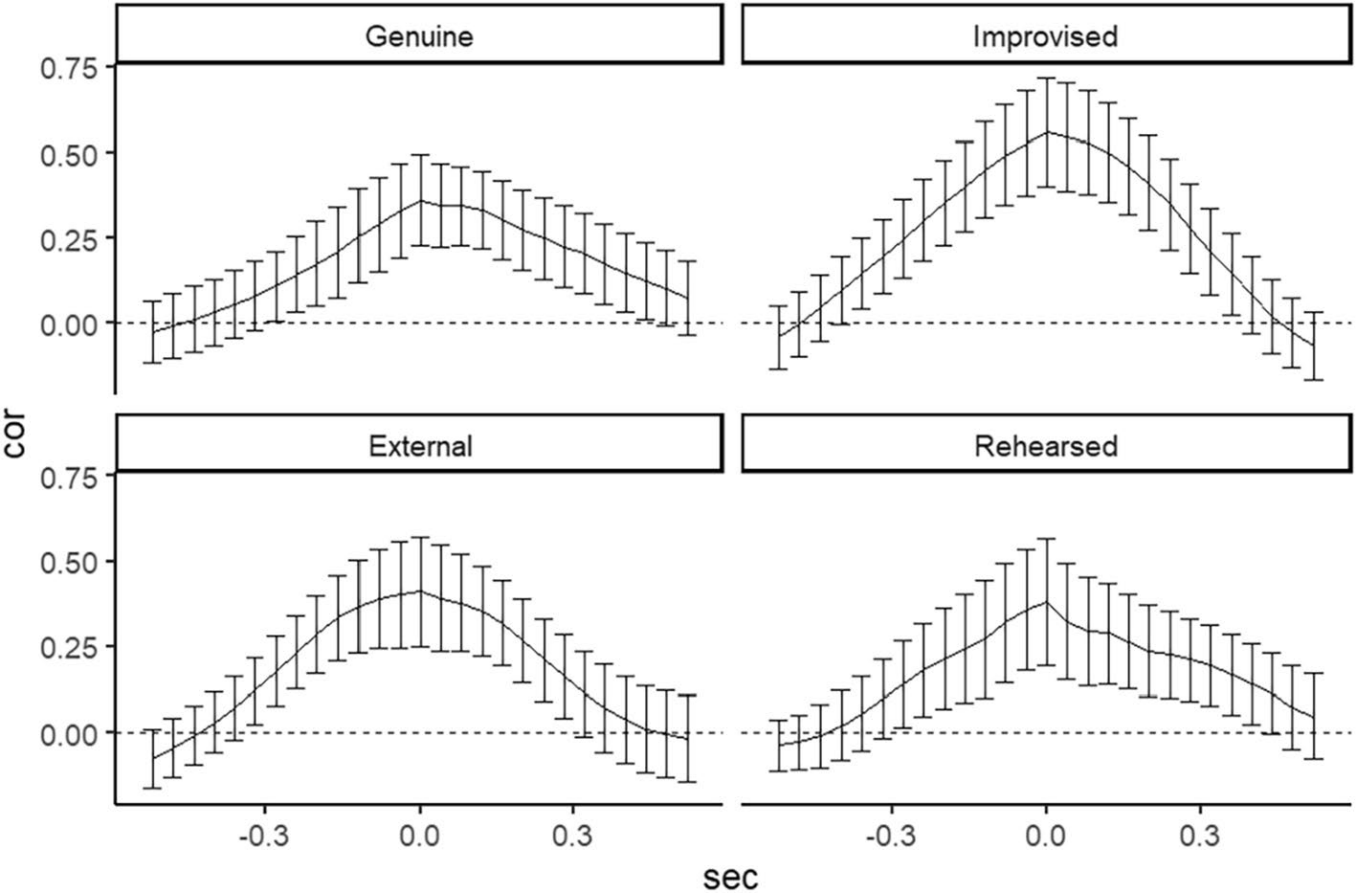
The cross-correlations between the right eyebrow and right eyelid for the four conditions. Error bar represents 95% Confidence Intervals.

As Fig. 6 represents the right parts of face, all conditions also indicated significant cross-correlations in accordance with the results of the left parts. This result also indicated the lack of follow-follower relationship between eyelid and eyebrow movement. The Improvised (*r* = 0.56, *SD* = 0.43, *95%CI* = [0.40, 0.72]) and External (*r* = 0.41, *SD* = 0.45, *95%CI* = [0.25, 0.57]) conditions showed medium couplings beyond zero at the lag 0 ms, and the pattern of coupling in the Rehearsed (*r* = 0.38, *SD* = 0.50, *95%CI* = [0.20, 0.57]) and Genuine (*r* = 0.36, *SD* = 0.37, *95%CI* = [0.23, 0.49]) conditions followed that. If participants had an experience of watching how the jack-in-the-box functions, the temporal coupling of eyelid and eyebrow movements was smaller than the other two deliberate expressions. Furthermore, the differences in coupling between conditions were stronger in the left parts than in the right.

## Discussion

The current study investigated whether genuine surprise displays differ from deliberate displays produced using varying methods (improvised, mimicked, or rehearsed) and the differences in their temporal features. The results indicated three key findings for the spatio-temporal features of surprise displays. First, ICA found that all surprise displays were mainly composed of raising eyebrows and eyelids movements, consistent with previous findings^33,50^ and that the left parts of genuine displays might include horizontal movements. Second, the speed of both eyebrows and eyelids when mimicking another person’s facial expressions (i.e., External condition) was generally faster than other displays. Finally, using cross-correlation disambiguated that there was no follow-follower relationship of eyelid and eyebrow movements, contrasting our predictions based on Namba et al.^10^. On the other hand, the Improvised deliberate displays showed the strongest coupling of these movements, and the genuine displays indicated the weakest coupling compared to the other displays. More interestingly, the Rehearsed deliberate displays also showed a lower cross-correlation than the other deliberate conditions.

ICA results clarified that raising both eyebrow and eyelid movements mainly contributed to all surprise displays. This makes sense, given that these movements are regarded as the main components of a surprise expression^1,31^. Considering that someone who is surprised wants to search for information to understand an unexpected situation (in this case, a jack-in-the-box), the facial movements related to opening the eyes such as raising the eyebrows and eyelids may function to gather more visual information^51^. As for Component 3, genuine displays were different from other displays (Fig. 2). According to Fig. 3, this component represents horizontal movement from only the left part of the face, and the result can be interpreted as genuine displays may contain asymmetrical movement. Although deliberate action has been often regarded as less symmetric rather than that which occurs spontaneously^52^, recent neuroanatomic observations supported left asymmetrical movement for spontaneous facial expressions compared to deliberate facial expressions^53^. This component may prove to be relevant in discriminating genuine from deliberate displays of surprise^8,19^.

For the speed of raising the eyebrows and eyelids, the deliberate facial display in the External condition was generally faster than other display conditions. In the External condition, expressers mimic a video from the Genuine condition. It should be noted that only participants in the External condition watched an actual spontaneous facial response of surprise. Such an observation of a facial reaction to the jack-in-the-box might create a representation of the surprised display in observers’ minds. The rapid deliberate displays produced when mimicking other’s facial responses may have been caused by the observed representation of a spontaneous surprise and/or rapid facial movements being generally perceived as more natural^54^. Indeed, Zloteanu et al.^19^ showed that the facial displays in the External condition were rated more genuine than the other deliberate displays. The facial movements of mimicking a surprise expression were quick, and the cause may be the misleading perception of quick eye and eyebrow movement being representative of spontaneous surprise.

The cross-correlation analysis in the current study provides empirical support for the idea that genuine surprise differs from deliberate displays with condition types (improvised, mimicked, or rehearsed) based on their temporal features. For all conditions, the correlation at lag 0 ms was the maximum value among all lags and there seemed to be no follow-follower relationship between the movements of eyelids and eyebrows, contrasting Namba et al.^10^ who reported eyelid movements preceded eyebrow movement in spontaneous surprise displays. Potentially, the sequential differences of eyebrow and eyelid movement may not be a robust property that distinguishes genuine from deliberate displays.

More interestingly, the strength of coupling between eyebrows and eyelids was largest in the surprise displays in the Improvised condition, followed in order by the External, Rehearsed, and Genuine condition. Accordingly, Zloteanu et al.^8^ found that observers’ genuineness rating to the Improvised displays is lower than the other facial displays. There may be a distinct co-occurrence of eyebrow and eyelid movements that is different from when expressing genuine surprise or when expressing deliberate surprise without any clues such as an example (video) or cause (jack-in-box). Additionally, the coupling between eyebrows and eyelids in the Rehearsed condition was smaller than in the other two deliberate displays and identical to genuine displays at least on the right parts of the face. According to motor simulation theory, motor representations/images are formed prior to execution^55^. In the Rehearsed condition, individuals might have been able to simulate motor representations of genuine human behavior to a jack-in-box by watching how it functions, leading to a behavioral coupling system similar to genuine displays. Further studies using a neuroanatomical approach are needed to determine if the actions of a stimulus like our jack-in-box can elicit such simulated actions.

While the current study showed that genuine surprise differs from deliberate displays, and deliberate displays also differ from each other based on their temporal features, several limitations should be noted. First, the current study does not include the self-reported feeling states of the expressers. Recent scholars discuss the importance of correspondence between facial displays and internal states^33^. For the Genuine condition, it can be assumed that the existence of participants who are not too surprised obscure the results. To address these gaps for genuine surprise displays, it is necessary to take first-person accounts of emotional experiences. However, there is also the issue that self-reported emotional experienced can only be indirectly measured because “qualia” can never be empirically measured^18,56^. Regardless, our results about speed and coupling indicated new evidence for the temporal features for surprise displays.

Second, several types of deliberate displays have not been treated in this research. For example, Porter^57^ investigated the morphological differences between not only spontaneous and simulated expressions but also masked and neutralized displays. A masked expression is when an expression corresponding to the felt emotion is replaced by a falsified expression that corresponds to a different emotion, while a neutralized expression is when the expression of a true emotion is inhibited and the face remains neutral^58^. These types of expressions should be considered in future extensions of temporal features of different deliberate displays research. Third, our use of between-participant designs, and unbalanced gender sampling should be noticed. The current study applied the between-participant design due to the nature of the task including unexpectedness, but such designs can introduce more variance (e.g., between behaviors and internal states) compared to within-participants design^59,60^. Although we confirmed that our results hold without the male data, we surmise that caution must be exercised when attempting to generalize from our sample. Thus, future studies will be necessary to investigate the temporal features of facial displays using within-participants design while considering gender as a potential factor. Finally, the current study tracked only 2-dimensional videos. Therefore, the complete horizontal movement (e.g., body movements) may ambiguate some other movements, such as the coupling between the left eyebrow and eyelid from the viewpoint. Indeed, ICA component 3 captured these from the Genuine condition. Of course, this can be interpreted as a specific feature of genuine surprise displays, but future studies should consider the depth information or target the 3-dimensional space to expand our understanding of how facial displays unfold over time and space.

Considering the initial question that asked how genuine surprise displays are different from deliberate surprise displays in terms of spatio-temporal properties, we attempted to provide an answer by combining several analytical methods, from tracking kinematics to temporal coupling. Together with comparisons of temporal features between genuine and deliberate displays above, the findings illustrate the complexity of the encoding aspects of human facial displays. Specifically, the left parts of genuine surprise might include horizontal movements, and the speed of both the eyebrow and eyelid when mimicking another person’s facial expressions was generally faster than for other displays. Moreover, the Improvised deliberate displays showed the strongest coupling of these movements, and the genuine displays indicated the weakest coupling compared to the other displays. Although we caution that the current study was exploratory in nature, future researchers will recognize the potential of temporal features as a locus for investigating the encoding aspect of facial displays. Accordingly, we hope that it stimulates further experimental investigation on decoding aspects of genuine versus deliberate facial displays.

## Supplementary Information

Supplementary Information.

## Data Availability

The datasets used in the current study are available from the corresponding author on reasonable request.
