# Supplementary material for: Distinct temporal features of genuine and deliberate facial expressions of surprise
Source: Sci Rep. 2021 Feb 9;11:3362. doi: 10.1038/s41598-021-83077-4 (PMC7873236; doi:10.1038/s41598-021-83077-4)
Supplement: Supplementary file 1 — Supplementary Information. [file 41598_2021_83077_MOESM1_ESM.docx]

**Supplemental information**

**Distinct temporal feature of genuine and deliberate facial expressions of surprise**

**Shushi Namba, Hiroshi Matsui, Mircea Zloteanu**

**Analysis of mouth area**

**ICA analysis for the mouth area**

We conducted a similar ICA analysis to confirm the mouth movements between conditions, following a similar procedure to that described the main paper. Fig S1 represented all tracked points on the mouth and topological information for each independent component. Four female participants in Genuine condition were excluded because she completely covered her mouth with her hands, resulting in 122 participants: Genuine condition = 28 (27 males 1 males), Improvised condition = 31 (28 females, 3 males), External condition = 32 (27 females 5 males), Rehearsed condition = 31 (28 females, 3 males). Results showed that the quantity and variety of mouth movements in participants facial reactions is too great to permit a reliable interpretation of each component using the mouth information (Fig1S, Fig2S).


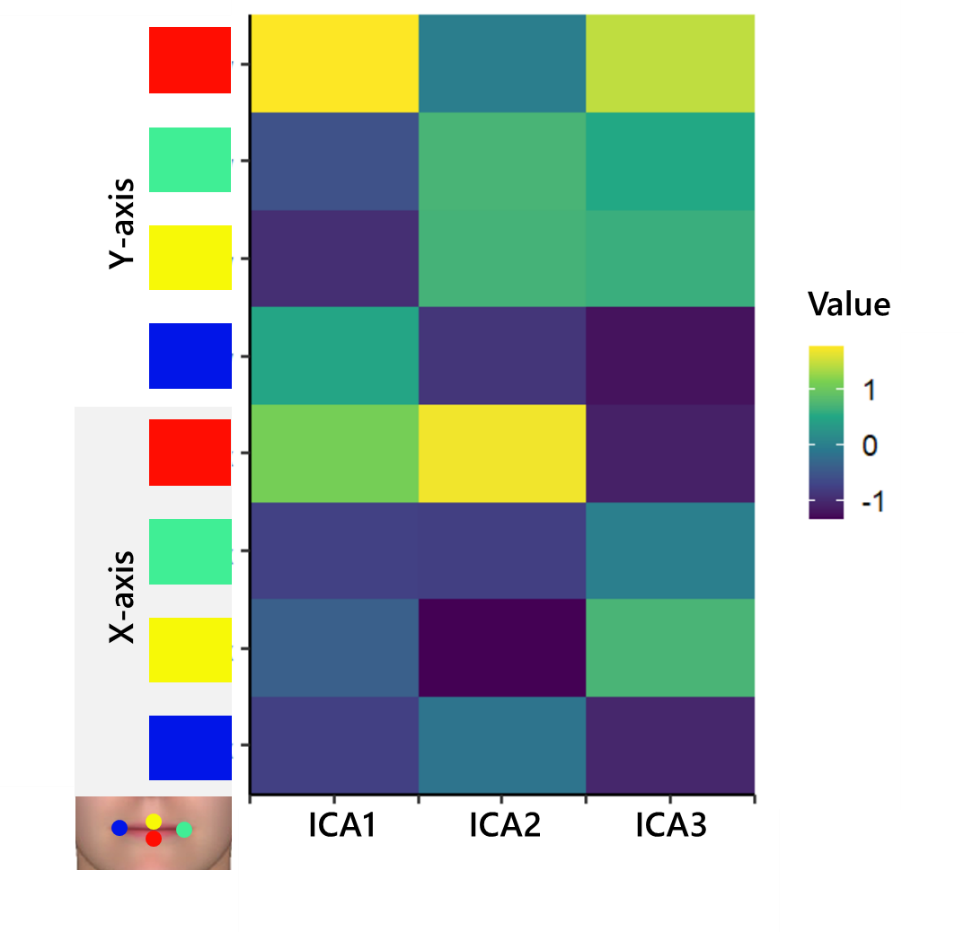


Figure S1. Heatmap of loadings for each Independent Component. Value colors represent the contribution of each facial part on independent component scores. Component 1 corresponded to the movement of the lower lip (Loading mean for the y-movements of the lower lip = 1.76; Loading mean for the x-movements for the lower lip = 1.10). Component 2 is the x-movement of upper and lower lips (Loading mean for the lower lip = 1.70; Loading mean for the lower lip = -1.32). Component 3 represents y-movements of the mouth (Loading mean for the lower lip = 1.45, Loading mean for the right lip corner = -1.21).


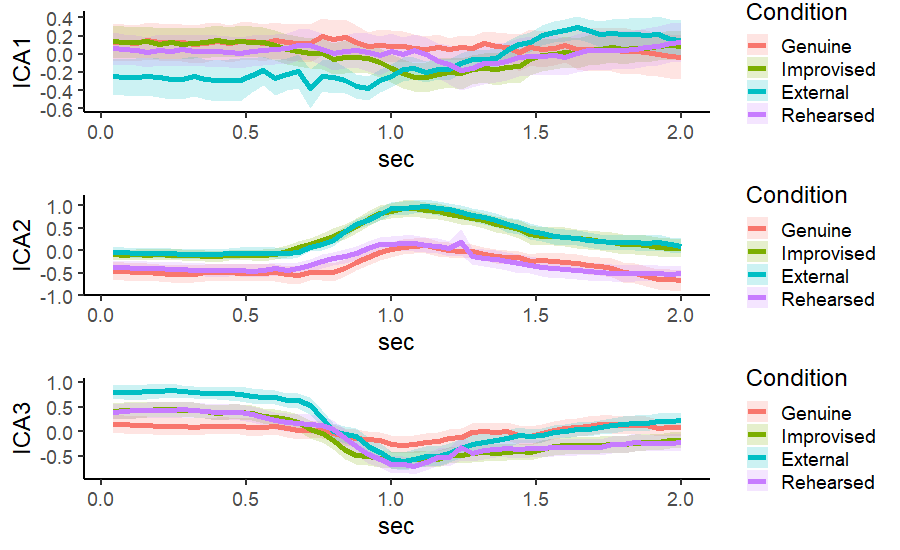


Figure S2. Independent Component among the four expression conditions. The ribbons represent ±1 standard error.

**Decreased size of opening of the mouth in genuine surprise**

To perform an interpretable mouth analysis, we calculated the difference between the y movements of the upper and lower lips. This index can be interpretable as the intensity of opening the mouth. From the visual inspection of Fig S3, the opening the mouth in the Genuine condition indicated a lower intensity than other conditions. At the peak point, there was a statistically significant effect of expression condition, *F*(3, 118) = 2.71, *p* = .049, $\eta_{G}^{2}$ = 0.06. In addition, multiple comparisons showed that the movement of opening the mouth in the Genuine condition was smaller than in the Improvised, *t*(59) = 2.75, *p* = .041, Hedge’s *g* = 0.70 [0.19, 1.22]; no other statistically significant comparisons were found, *t*s < 2.07, *p*s > 0.12, Hedge’s *g*s < .053.


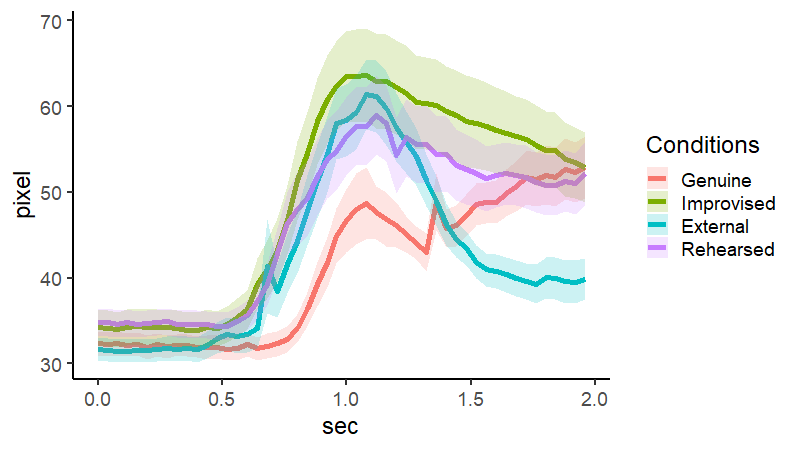


Figure S3. The intensity of opening the mouth among the four expression conditions. The ribbons represent ±1 standard error.
